# The impact of pre-transplantation diabetes and obesity on acute graft-versus-host disease, relapse and death after allogeneic hematopoietic cell transplantation: a study from the EBMT Transplant Complications Working Party

**DOI:** 10.1038/s41409-023-02154-6

**Published:** 2023-12-07

**Authors:** Lars Klingen Gjærde, Tapani Ruutu, Christophe Peczynski, William Boreland, Nicolaus Kröger, Didier Blaise, Thomas Schroeder, Régis Peffault de Latour, Tobias Gedde-Dahl, Aleksandr Kulagin, Henrik Sengeløv, Ibrahim Yakoub-Agha, Jürgen Finke, Matthias Eder, Grzegorz Basak, Ivan Moiseev, Hélène Schoemans, Christian Koenecke, Olaf Penack, Zinaida Perić

**Affiliations:** 1 Department of Hematology, Rigshospitalet, Copenhagen University Hospital, Copenhagen, Denmark; 2 Clinical Research Institute, Helsinki University Hospital, Helsinki, Finland; 3 EBMT Paris Study Office, Paris, France; 4 University Medical Center Hamburg, Hamburg, Germany; 5 Institut Paoli Calmettes, Marseille, France; 6 University Hospital, Essen, Germany; 7 Hopital St. Louis, Paris, France; 8 Oslo University Hospital, Rikshospitalet, Oslo, Norway; 9 First State Pavlov Medical University of St. Petersburg, St., Petersburg, Russia; 10 Université de Lille, Lille, France; 11 University of Freiburg, Freiburg, Germany; 12 Hannover Medical School, Hannover, Germany; 13 University Clinical Center of the Medical University of Warsaw, Warsaw, Poland; 14 KU Leuven-University of Leuven, Leuven, Belgium; 15 Charité-Universitätsmedizin Berlin, Berlin, Germany; 16 University of Zagreb, Zagreb, Croatia; 17 University Hospital Centre Zagreb, Zagreb, Croatia

**Keywords:** Risk factors, Graft-versus-host disease, Epidemiology

## Abstract

Obesity and diabetes can modulate immune responses, which may impact allogeneic HCT outcomes and GvHD. From the EBMT registry, we included 36,539 adult patients who underwent allogeneic HCT for a hematological malignancy between 2016 and 2020. Of these, 5228 (14%) had obesity (BMI ≥ 30 kg/m^2^), 1415 (4%) had diabetes (requiring treatment with insulin or oral hypoglycemics), and 688 (2%) had obesity + diabetes pre-transplantation. Compared with patients without diabetes or obesity, the hazard ratio (HR) of grade II–IV acute GvHD was 1.00 (95% confidence interval [CI] 0.94–1.06, *p* = 0.89) for patients with obesity, 0.95 (CI 0.85–1.07, *p* = 0.43) for patients with diabetes, and 0.96 (CI 0.82–1.13, *p* = 0.63) for patients with obesity + diabetes. Non-relapse mortality was higher in patients with obesity (HR 1.08, CI 1.00–1.17, *p* = 0.047), diabetes (HR 1.40, CI 1.24–1.57, *p* < 0.001), and obesity + diabetes (HR 1.38, CI 1.16–1.64, *p* < 0.001). Overall survival after grade II–IV acute GvHD was lower in patients with diabetes (HR 1.46, CI 1.25–1.70, *p* < 0.001). Pre-transplantation diabetes and obesity did not influence the risk of developing acute GvHD, but pre-transplantation diabetes was associated with poorer survival after acute GvHD.

## Introduction

Obesity and diabetes can induce metabolic changes that modulate immune responses and promote a chronic low-grade inflammatory state [1, 2]. In the context of allogeneic hematopoietic cell transplantation (HCT), pre-clinical studies have found that obese mice develop more severe acute graft-versus-host disease (GvHD) after allogeneic HCT, possibly due to an increased pro-inflammatory cytokine production and a dysregulation of the gut microbiome [3, 4]. Likewise, post-transplant hyperglycemia and new-onset post-transplantation diabetes have been shown to increase the risk of acute GvHD [5–7], possibly due to an exaggeration of the inflammatory response and a dysregulation of immunological signaling and endothelial function [8–11].

In observational studies of patients undergoing allogeneic HCT, associations between obesity and pre-transplantation diabetes and acute GvHD have been less clear. Regarding obesity, a Japanese registry study of 3827 adult patients found an increased risk of developing grade II–IV acute GvHD when having a pre-transplant body-mass index (BMI) ≥ 30 kg/m^2^ compared with having a BMI between 18 and 25 kg/m^2^, but this did not remain statistically significant in multivariable analysis [12]. Other registry studies have not found support for an association between BMI and acute GvHD [13–16]. Likewise, studies of pediatric transplant cohorts have been conflicting [17–20]. A more consistent association, however, has been shown between obesity and non-relapse mortality (NRM) in general [13, 14, 21–27].

Regarding pre-transplantation diabetes, two larger studies from Japan and the US, which included 378 and 134 patients, respectively, with pre-transplantation diabetes did not find support for a difference in the risk of acute GvHD when compared with patients without pre-transplantation diabetes [28, 29]. The Japanese study did however find a higher cumulative incidence of GvHD-related mortality at 1-year in patients with pre-transplantation diabetes (3.6% vs. 2.0%, *p* = 0.05). Still, all patients included in the two studies were transplanted before 2010; hence, only a relatively small proportion of patients underwent a reduced intensity/non-myeloablative transplant, which in later years has been increasingly used in older patients and in patients with significant comorbidities, including diabetes and obesity.

A recent study from the European Society for Blood and Marrow Transplantation (EBMT) found that in patients transplanted for a hematological malignancy from a matched sibling or unrelated donor between 2010 and 2018, 4.4% had pre-transplantation diabetes, defined as diabetes requiring treatment with insulin or oral hypoglycemics but not diet alone, and 3.9% had pre-transplant obesity, defined as having a BMI ≥ 35 kg/m^2^ [23]. Given the low prevalence of pre-transplantation diabetes and obesity, a large registry study is required to yield conclusive estimates of the impact of obesity and diabetes on acute GvHD and acute GvHD-related mortality in recent years. This is needed to improve the prognostic information given to future patients undergoing allogeneic HCT with pre-existing diabetes or obesity, two patient groups that are likely to increase in numbers over the coming years [30, 31].

## Patients and methods

### Study design

We conducted a retrospective observational study using data from the EBMT registry. We included all adult (≥18 years of age) patients, who underwent their first (previous autologous transplantations were allowed) bone marrow or peripheral blood stem cell allogeneic transplantation for a hematological malignancy between 2016 and 2020, and who had complete information on acute GvHD status or grade and on height, weight, and diabetes comorbidity prior to transplant. Exclusion criteria included ex vivo T-cell depletion and donor types other than identical sibling, matched unrelated, mismatched unrelated or haploidentical donor. The study was planned and approved by the Transplant Complications Working Party of the EBMT. All patients gave written informed consent to use their personal data for research purposes. The study was conducted in accordance with the Declaration of Helsinki.

Patients with pre-transplantation obesity, diabetes, and obesity + diabetes, respectively, were compared with patients without pre-transplantation obesity and diabetes (control group). The primary objective was to compare the incidence of grade II–IV acute GvHD. The secondary objectives included comparing the incidence of chronic GvHD, overall survival (OS), relapse, NRM, and OS after grade II–IV acute GvHD between the groups of interest.

### Definitions

In 2016, pre-existing comorbidities were added to the EBMT Minimum Essential Data (MED)-A form, a mandatory form to submit to the EBMT for all allogeneic HCTs in all EBMT centers. We defined cases of pre-transplantation diabetes using the MED-A form’s Comorbidity Index item “Diabetes” (defined as “requiring treatment with insulin or oral hypoglycemics, but not diet alone”), and obesity as having a BMI ≥ 30 kg/m^2^ calculated from the MED-A form’s information on pre-transplantation weight and height. Acute GvHD was defined and graded based on the Keystone criteria [32]. Chronic GvHD was defined and classified as limited or extensive according to published criteria [33]. OS was defined as death from any cause, and NRM was defined as death without relapse.

### Statistical analyses

Start time was the date of transplant for all endpoints, except for the secondary endpoint of OS after a diagnosis of grade II–IV acute GvHD. For this specific endpoint, start date was the date of the first occurrence of grade II–IV acute GvHD.

Probabilities of OS were calculated using the Kaplan–Meier estimator. Cumulative incidence functions were used to estimate NRM and relapse incidence in a competing risk setting, death and relapse competing with each other. For the estimation of the cumulative incidence of acute and chronic GvHD, relapse and death were considered to be competing events. For the analysis of OS after acute GvHD grade II–IV, we performed a landmark analysis (starting from the time of first occurrence) of all patients who experienced grade II–IV acute GvHD before day +180.

Hazard ratios (HR), together with the 95% confidence intervals (CI) and *p*-values were estimated using multivariable cause-specific Cox proportional hazards regression models, providing a cause-specific HR in the presence of competing risks. To adjust the HRs for the comparison between the groups of interest, the following covariates were included: previous autologous transplantation (yes vs. no), year of transplantation (continuous), cell source (bone marrow vs. peripheral blood), type of donor (identical sibling vs. matched unrelated donor vs. mismatched unrelated donor vs. haploidentical donor), Disease Risk Index (high-very high vs. low-intermediate), patient age (continuous), patient and donor sex (male vs. female), patient and donor CMV (negative vs. positive), Karnofsky score (≥90 vs. <90), intensity of conditioning (RIC vs. MAC), TBI (yes vs. no), and use of ATG/Campath (yes vs. no). For the landmark analysis of OS after acute GvHD grade II–IV, grade of acute GvHD and time between transplantation and acute GvHD (logarithmic transformation of the continuous time) were also added to the covariate set. These variables were selected because of known clinical relevance, impact in univariable analysis and/or statistically significant imbalance between the groups of interest.

Differences were considered statistically significant in case of *p* < 0.05. Center effect was taken into account by introducing a random effect or ‘frailty’ into all models. Statistical analyses were performed using R version 4.1.2 (R Foundation for Statistical Computing, Vienna, Austria).

## Results

### Patient characteristics and prevalence of pre-transplantation diabetes and obesity

A total of 36 539 patients were included, of which 5228 (14%) had obesity (without co-existing diabetes), 1415 (4%) had diabetes (without co-existing obesity), and 688 (2%) had obesity + diabetes prior to transplant. Table 1 shows the patient characteristics according to pre-transplantation diabetes and obesity. The majority of patients received a peripheral blood stem cell transplant (89.5% of the control group, 92.6% of patients with obesity, 92.2% of patients with diabetes, and 96.6% of patients with obesity + diabetes). The most common donor was a 10/10 HLA-matched unrelated donor (used in 40.4% of the control group, 40.9% of patients with obesity, 41.4% of patients with diabetes, and 44.9% of patients with obesity + diabetes), and the most common indication was acute myeloid leukemia (58.8% of the control group, 57.2% of patients with obesity, 55.6% of patients with diabetes, and 54.4% of patients with obesity + diabetes). Reduced intensity conditioning was used in 49.2% of the control group, 51.5% of patients with obesity, 61.4% of patients with diabetes, and 63.4% of patients with obesity + diabetes.

**Table 1:** Patient characteristics according to pre-transplantation diabetes and obesity.

Characteristic	Control group (*N* = 29,208)	Obesity (*N* = 5228)	Diabetes (*N* = 1415)	Obesity + Diabetes (*N* = 688)
Patient age in years, median (min-max) [IQR]	54.3 (18–82.5) [41.4–62.7]	53.8 (18–76.8) [43.2–61.5]	61.5 (18–83.8) [55.1–66.4]	59.5 (20.8–76.2) [53.8–64.2]
Patient sex, *N* (%)				
Male	17,062 (58.5%)	3027 (57.9%)	1007 (71.2%)	451 (65.7%)
Female	12118 (41.5%)	2198 (42.1%)	407 (28.8%)	235 (34.3%)
Missing	28	3	1	2
Karnofsky score, *N* (%)				
≥90	20,208 (72.3%)	3672 (73.4%)	825 (61.6%)	433 (65.8%)
<90	7752 (27.7%)	1331 (26.6%)	514 (38.4%)	225 (34.2%)
Missing	1248	225	76	30
HCT-CI, *N* (%)				
0	16,769 (59.4%)	2243 (44.6%)	0 (0%)	0 (0%)
1–2	5310 (18.8%)	1700 (33.8%)	630 (51%)	298 (51.3%)
3+	6134 (21.7%)	1090 (21.7%)	605 (49%)	283 (48.7%)
Missing	995	195	180	107
Renal comorbidity (moderate/severe)^a^, *N* (%)				
Yes	319 (1.1%)	72 (1.4%)	45 (3.4%)	22 (3.5%)
No	28736 (98.9%)	5130 (98.6%)	1271 (96.6%)	608 (96.5%)
Missing	153	26	99	58
Cardiac comorbidity^b^, *N* (%)				
Yes	1531 (5.3%)	309 (5.9%)	217 (16.3%)	104 (16.3%)
No	27,548 (94.7%)	4896 (94.1%)	1117 (83.7%)	534 (83.7%)
Missing	129	23	81	50
Cerebrovascular disease^c^, *N* (%)				
Yes	458 (1.6%)	97 (1.9%)	56 (4.3%)	16 (2.6%)
No	28,639 (98.4%)	5102 (98.1%)	1255 (95.7%)	608 (97.4%)
Missing	111	29	104	64
Body mass index in kg/m^2^, median (min-max) [IQR]	24.2 (12.9–30) [21.9–26.5]	32.5 (30–65.9) [31.1–34.9]	25.6 (12.1–30) [23.5–27.7]	32.9 (30–55.1) [31.2–35.6]
Diagnosis, *N* (%)				
Acute leukemia	17,183 (58.8%)	2989 (57.2%)	787 (55.6%)	374 (54.4%)
Chronic leukemia	1176 (4%)	266 (5.1%)	55 (3.9%)	29 (4.2%)
Lymphoma	3668 (12.6%)	629 (12%)	128 (9%)	65 (9.4%)
Plasma cell disorders	792 (2.7%)	182 (3.5%)	37 (2.6%)	22 (3.2%)
MDS/MPN	6389 (21.9%)	1162 (22.2%)	408 (28.8%)	198 (28.8%)
Disease risk index, *N* (%)				
Low	2519 (9.2%)	560 (11.4%)	94 (7.1%)	48 (7.5%)
Intermediate	17,315 (63.1%)	3139 (63.7%)	832 (63.1%)	416 (65.1%)
High	6428 (23.4%)	1060 (21.5%)	332 (25.2%)	155 (24.3%)
Very High	1165 (4.2%)	170 (3.4%)	60 (4.6%)	20 (3.1%)
Missing	1781	299	97	49
Donor type, *N* (%)				
Identical sibling	9034 (30.9%)	1652 (31.6%)	406 (28.7%)	211 (30.7%)
MUD 10/10	11,797 (40.4%)	2140 (40.9%)	586 (41.4%)	309 (44.9%)
MMUD 9/10	2968 (10.2%)	541 (10.3%)	137 (9.7%)	75 (10.9%)
MMUD 8/10 or less	383 (1.3%)	80 (1.5%)	16 (1.1%)	12 (1.7%)
Haploidentical	5026 (17.2%)	815 (15.6%)	270 (19.1%)	81 (11.8%)
Cell source, *N* (%)				
Bone marrow	3071 (10.5%)	385 (7.4%)	110 (7.8%)	21 (3.1%)
Peripheral blood	26,137 (89.5%)	4843 (92.6%)	1305 (92.2%)	667 (96.9%)
Conditioning intensity, *N* (%)				
Myeloablative	14,716 (50.8%)	2530 (48.9%)	540 (38.6%)	247 (36.6%)
Reduced intensity	14,237 (49.2%)	2646 (51.1%)	858 (61.4%)	427 (63.4%)
Missing	255	52	17	14
Total-body irradiation, *N* (%)	6425 (22%)	1090 (20.8%)	254 (18%)	126 (18.3%)
In vivo T-cell depletion				
ATG/Campath	16,151 (55.7%)	3061 (58.9%)	816 (58%)	424 (61.9%)
No	12,858 (44.3%)	2140 (41.1%)	591 (42%)	261 (38.1%)
Missing	199	27	8	3

*ATG* anti-thymocyte globulin, *HCT-CI* hematopoietic cell transplantation-specific comorbidity index, *IQR* inter-quartile range, *MDS* myelodysplastic syndrome, *MPN* myeloproliferative neoplasm, *(M)MUD* (mis)matched unrelated donor.

^a^Defined from the HCT-CI as having serum creatinine >2 mg/dL (>177 μmol/L), on dialysis, or prior renal transplantation.

^b^Defined from the HCT-CI as having coronary artery disease, congestive heart failure, myocardial infarction, or ejection fraction ≤ 50%.

^c^Defined from the HCT-CI as having had a transient ischemic attack or cerebrovascular accident.

With regards to clinically relevant differences between the groups of interest, patients with diabetes and obesity + diabetes were older (median [inter-quartile range (IQR)] age 61.5 [55.1–66.4] years and 59.5 [53.8–64.2] years, respectively) than patients with obesity and the control group (median [IQR] age 53.8 [43.2–61.5] years and 54.3 [41.4–62.7] years, respectively), and they were more likely to be male (71.2% and 65.7% males in patients with diabetes and obesity + diabetes, respectively, versus 57.9% and 58.5% males in patients with obesity and the control group, respectively), and have a Karnofsky performance score <90 (38.3% and 34.2% of patients with diabetes and obesity + diabetes, respectively, versus 26.6% and 27.7% of patients with obesity and the control group, respectively).

The median follow-up was 32.7 months in the control group, 31.0 months in patients with obesity, 32.6 months in patients with diabetes, and 31.9 months in patients with obesity + diabetes.

### Acute GvHD

**Fig. 1:**
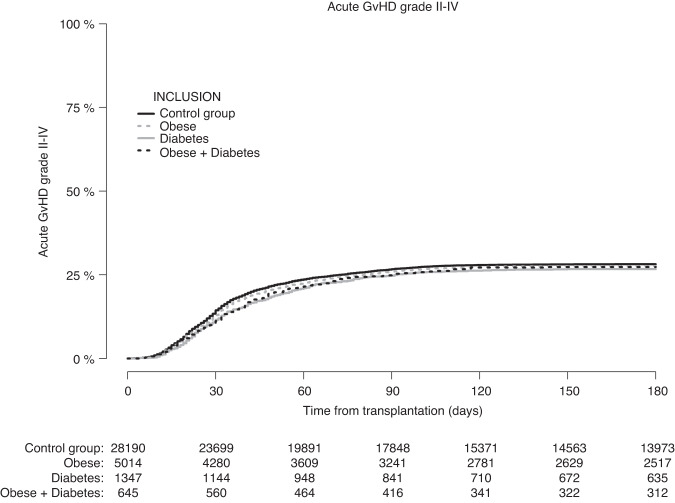
Patients with missing data on acute GvHD status were excluded. Cumulative incidence of grade II–IV acute graft-versus-host disease according to pre-transplantation obesity and diabetes status.

Figure 1 shows the cumulative incidence of grade II–IV acute GvHD according to pre-transplantation obesity and diabetes status. At day +100, the cumulative incidence of grade II–IV acute GvHD was 26.6% (CI: 25.4–27.9%) in patients with obesity, 25.7% (CI: 23.4–28.0%) in patients with diabetes, 25.8% (CI: 22.5–29.2%) in patients with obesity + diabetes, compared with 27.3% (CI: 26.8–27.9%) in the control group of patients without pre-transplantation obesity and diabetes. Similar results were seen at day +180 (Table 2).

**Table 2:** Cumulative incidence [with 95% confidence interval] of acute and chronic GvHD, NRM, relapse, and OS according to pre-transplantation obesity and diabetes status.

Outcome	Control group (*N* = 29,208)	Obesity (*N* = 5228)	Diabetes (*N* = 1415)	Obesity + Diabetes (*N* = 688)
Acute GvHD grade II–IV (at day +100)	27.3% [26.8–27.9]	26.6% [25.4–27.9]	25.7% [23.4–28.0]	25.8% [22.5–29.2]
Chronic GvHD, limited and extensive (at 2 years)	34.4% [33.8–35.0]	34.9% [33.5–36.3]	33.0% [30.4–35.7]	36.6% [32.6–40.6]
Extensive chronic GvHD (at 2 years)	15.8% [15.3–16.2]	15.2% [14.1–16.3]	15.3% [13.3–17.5]	19.0% [15.8–22.4]
NRM (at 1-year)	14.0% [13.6–14.4]	15.5% [14.5–16.5]	23.9% [21.6–26.3]	20.0% [16.9–23.3]
NRM (at 2 years)	16.8% [16.4–17.3]	18.2% [17.1–19.4]	27.9% [25.4–30.4]	24.6% [21.1–28.2]
Relapse (at 1-year)	22.8% [22.3–23.3]	21.8% [20.6–23.0]	23.1% [20.8–25.4]	20.7% [17.5–24.1]
Relapse (at 2 years)	28.7% [28.2–29.3]	28.2% [26.9–29.6]	28.4% [25.8–31.0]	27.7% [24.0–31.5]
OS (at 1-year)	73.2% [72.7–73.7]	73.0% [71.8–74.3]	61.5% [59.0–64.2]	66.9% [63.4–70.7]
OS (at 2 years)	63.6% [63.0–64.2]	63.9% [62.5–65.3]	50.1% [47.4–53.0]	55.0% [51.1–59.2]

*GvHD* graft-versus-host disease, *OS* overall survival, *NRM* non-relapse mortality.

In multivariable analysis, the adjusted HR of grade II–IV acute GvHD—using the control group as reference—was 1.00 (CI: 0.94–1.06, *p* = 0.89) for patients with obesity, 0.95 (CI: 0.85–1.07, *p* = 0.43) for patients with diabetes, and 0.96 (CI: 0.82–1.13, *p* = 0.63) for patients with obesity + diabetes (Table 3).

**Table 3:** Estimates of the impact of pre-transplantation obesity and diabetes on grade II–IV acute graft-versus-host disease and other transplant outcomes from multivariable cause-specific Cox proportional hazard regression models.

Outcome	Reference: Control group	Adjusted^a^ HR [95% CI]	*P*
Grade II–IV acute GvHD	Obesity	1.00 [0.94–1.06]	0.89
	Diabetes	0.95 [0.85–1.07]	0.43
	Obesity + Diabetes	0.96 [0.82–1.13]	0.63
Chronic GvHD (limited + extensive)	Obesity	1.04 [0.98–1.10]	0.17
	Diabetes	1.02 [0.92–1.14]	0.68
	Obesity + Diabetes	1.07 [0.93–1.25]	0.34
Extensive chronic GvHD	Obesity	1.00 [0.92–1.08]	0.92
	Diabetes	1.07 [0.92–1.25]	0.37
	Obesity + Diabetes	1.18 [0.97–1.44]	0.10
Overall survival	Obesity	1.02 [0.97–1.08]	0.38
	Diabetes	1.29 [1.18–1.40]	<0.0001
	Obesity + Diabetes	1.23 [1.09–1.39]	0.001
Relapse	Obesity	1.02 [0.96–1.08]	0.53
	Diabetes	1.08 [0.96–1.20]	0.20
	Obesity + Diabetes	0.96 [0.81–1.13]	0.61
Non-relapse mortality	Obesity	1.08 [1.00–1.17]	0.047
	Diabetes	1.40 [1.24–1.57]	<0.0001
	Obesity + Diabetes	1.38 [1.16–1.64]	0.0003
Overall survival after grade II–IV acute GvHD	Obesity	1.02 [0.93–1.13]	0.65
	Diabetes	1.46 [1.25–1.70]	<0.0001
	Obesity + Diabetes	1.18 [0.95–1.47]	0.14

For the landmark analysis of OS after acute GvHD grade II–IV, grade of acute GvHD and time between transplantation and acute GvHD (logarithmic transformation of the continuous time) were also added to the covariate set.

^a^The following covariates were included: previous autologous transplantation (yes vs. no), year of transplantation (continuous), cell source (bone marrow vs. peripheral blood), type of donor (identical sibling vs. matched unrelated donor vs. mismatched unrelated donor vs. haploidentical donor), Disease Risk Index (high-very high vs. low-intermediate), patient age (continuous), patient and donor sex (male vs. female), patient and donor CMV (negative vs. positive), Karnofsky score (≥90 vs. < 90), intensity of conditioning (RIC vs. MAC), TBI (yes vs. no), and use of ATG/Campath (yes vs. no).

*CI* confidence interval, *GvHD* graft-versus-host disease, *HR* hazard ratio.

### Chronic GvHD

The 2-year cumulative incidence of chronic GvHD (both limited and extensive) was 34.9% (CI: 33.5–36.3%) in patients with obesity, 33.0% (CI: 30.4–35.7%) in patients with diabetes, 36.6% (CI: 32.6–40.6%) in patients with obesity + diabetes, compared with 34.4% (CI: 33.8–35.0%) in the control group. The 2-year cumulative incidence of extensive chronic GvHD was 15.2% (CI: 14.1–16.3%) in patients with obesity, 15.3% (CI: 13.3–17.5%) in patients with diabetes, 19.0% (CI: 15.8–22.4%) in patients with obesity + diabetes, compared with 15.8% (CI: 15.3–16.2%) in the control group.

The adjusted HR, compared with the control group, for chronic GvHD (limited and extensive) was 1.04 (CI: 0.98–1.10, *p* = 0.17) for patients with obesity, 1.02 (CI: 0.92–1.14, *p* = 0.68) for patients with diabetes, and 1.07 (CI: 0.93–1.25, *p* = 0.34) for patients with obesity + diabetes. For extensive chronic GvHD alone, the adjusted HR was 1.00 (CI: 0.92–1.08, *p* = 0.92) for patients with obesity, 1.07 (CI: 0.92–1.25, *p* = 0.37) for patients with diabetes, and 1.18 (CI: 0.97–1.44, *p* = 0.10) for patients with obesity + diabetes (Table 3).

### NRM, relapse and OS

**Fig. 2:**
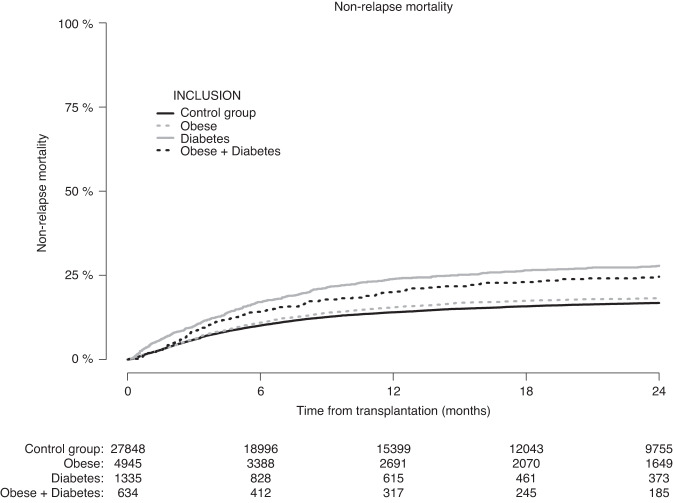
Patients with missing data on relapse status were excluded. Non-relapse mortality according to pre-transplantation obesity and diabetes status.

Figure 2 shows the incidence of NRM according to pre-transplantation obesity and diabetes status. The 1-year NRM was 14% (CI: 13.6–14.4%) in the control group, 15.5% (CI: 14.5–16.5%), in patients with obesity, 23.9% (CI: 21.6–26.3%) in patients with diabetes, and 20.0% (CI: 16.9–23.3%) in patients with obesity + diabetes. Similar differences were observed at 2-years (Table 2). In multivariable analysis, the adjusted HR (using the control group as reference) for NRM was 1.08 (CI: 1.00–1.17, *p* = 0.047) for patients with obesity, 1.40 (CI: 1.24–1.57, *p* < 0.0001) for patients with diabetes, and 1.38 (CI: 1.16–1.64, *p* = 0.0003) for patients with obesity + diabetes (Table 3).

**Fig. 3:**
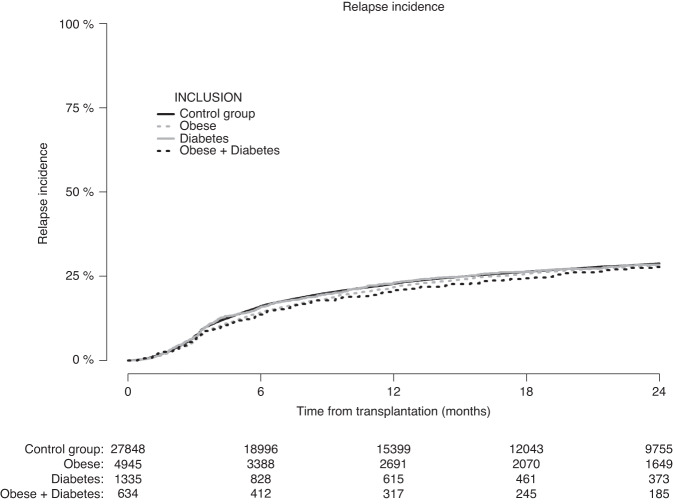
Patients with missing data on relapse status were excluded. Cumulative incidence of relapse according to pre-transplantation obesity and diabetes status.

The cumulative incidence of relapse (Fig. 3) at 2-years was 28.2% (CI: 26.9–29.6%) in patients with obesity, 28.4% (CI: 25.8–31.0%) in patients with diabetes, and 27.7% (CI: 24.0–31.5%) in patients with obesity + diabetes, compared with 28.7% (CI: 28.2–29.3%) in the control group. The adjusted HR for relapse was 1.02 (CI: 0.96–1.08, *p* = 0.53) for patients with obesity, 1.08 (CI: 0.96–1.20, *p* = 0.20) for patients with diabetes, and 0.96 (CI: 0.81–1.13, *p* = 0.61) for patients with obesity + diabetes (Table 3).

**Fig. 4:**
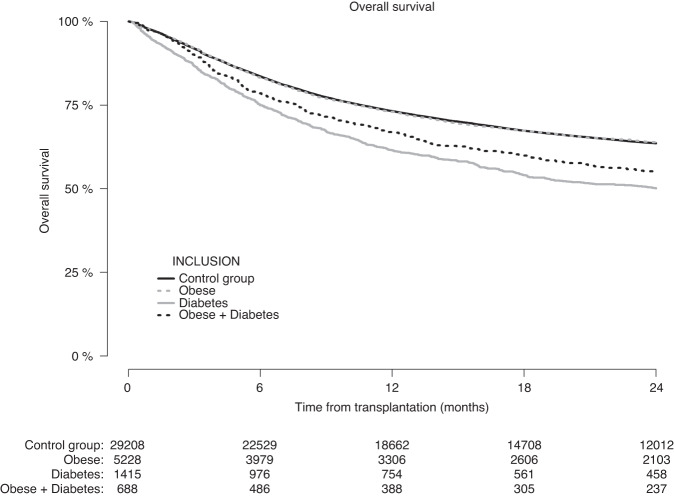
Overall survival after allogeneic hematopoietic cell transplantation according to pre-transplantation obesity and diabetes status.

OS (Fig. 4) at 2-years was 63.9% (CI: 62.5–65.3%) in patients with obesity, 50.1% (CI: 47.4–53.0%) in patients with diabetes, and 55.0% (CI: 51.1–59.2%) in patients with obesity + diabetes, compared with 63.3% (CI: 63.0–64.2%) in the control group. The adjusted HR for OS was 1.02 (CI: 0.97–1.08, *p* = 0.38) for patients with obesity, 1.29 (CI: 1.18–1.40, *p* < 0.0001) for patients with diabetes, and 1.23 (CI: 1.09–1.39, *p* = 0.001) for patients with obesity + diabetes (Table 3).

### Survival after acute GvHD

**Fig. 5:**
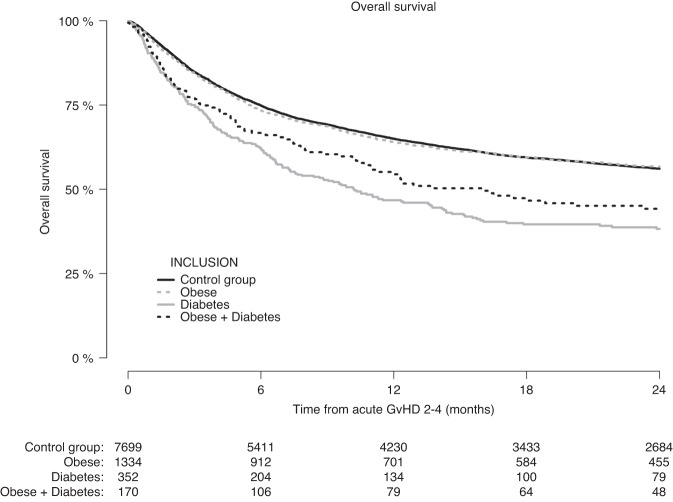
Only patients who experienced grade II-IV acute GvHD within day +180 were included. Overall survival after diagnosis of grade II–IV acute graft-versus-host disease according to pre-transplantation obesity and diabetes status.

Figure 5 shows the OS according to pre-transplantation obesity and diabetes from the date of first occurrence of grade II–IV acute GvHD in the group of patients who experienced this within the first 180 days after allogeneic HCT (*N* = 9555). At 24-months after the first occurrence of grade II–IV acute GvHD, the OS of patients with pre-transplantation obesity (56.7%, CI: 54.0–59.6%) was similar to that in the control group (56.1%, CI: 54.9–57.3%), whereas patients with pre-transplantation diabetes and patients with pre-transplantation obesity + diabetes had a lower OS of 38.3% (CI: 33.2–44.1%) and 43.3% (CI: 36.1–52.1%), respectively.

In multivariable analysis (Table 3), having pre-transplantation diabetes was associated with poorer OS after a diagnosis of grade II–IV acute GvHD compared to patients without pre-transplantation diabetes or obesity (HR 1.46, CI: 1.25–1.70, *p* < 0.0001). We did not find support for an inferior survival—in comparison to the control group—in patients with pre-transplantation obesity (HR 1.02, CI: 0.93–1.13, *p* = 0.65) or with pre-transplantation obesity + diabetes (HR 1.18, CI: 0.95–1.47, *p* = 0.14).

## Discussion

We found that patients with pre-transplantation diabetes, obesity, or obesity + diabetes did not have an increased risk of developing grade II–IV acute GvHD compared to patients without pre-transplantation diabetes or obesity. Likewise, we did not find support for any difference in the risk of chronic GvHD. However, NRM was higher in patients with pre-transplantation diabetes, obesity or obesity+diabetes, respectively, compared to the control group. Moreover, in patients who experienced grade II–IV acute GvHD, the OS from the diagnosis of GvHD was lower in patients with pre-transplantation diabetes. Our finding that pre-existing obesity and diabetes do not influence the risk of acute GvHD after allogeneic HCT is in agreement with previous studies [12–16, 28, 29]. Our findings extend those of the previous studies by having studied the association in a large and more recent transplant cohort, in which a significant proportion (50.2% in total) of patients received reduced-intensity conditioning.

At the same time, however, our study—as well as several previous studies [13, 14, 21–27]—found a strong association between pre-transplantation obesity and diabetes and an increased risk of NRM. Since the risk of acute GvHD was not influenced by obesity and diabetes, the increased NRM is likely to be driven by an increased risk of conditioning-induced organ damage and infections. While we did not have data on cause of death in sufficient detail nor on the incidence of infections to analyze this, previous cohort studies from Japan have established that pre-transplantation obesity entails a higher risk of systemic infections [12] and that pre-transplantation diabetes, in multivariable analysis, was associated with an increased risk of mucor infection (but not infection overall) and infection- and organ failure-related NRM [28]. The latter study also found an increase in the 1-year GvHD-related mortality in patients with pre-transplantation diabetes [28]; This echoes our finding of a decreased OS after acute GvHD in patients with pre-transplantation diabetes; a potential cause may be that patients with diabetes have more dysglycemia following steroid treatment for acute GvHD, which has been associated with lower survival [34]. While the survival after grade II–IV acute GvHD was numerically inferior also in those with obesity + diabetes, this was not statistically significant in multivariable analysis. This finding could be an artifact of lower power or that patients with obesity + diabetes differ from those with diabetes without obesity in ways (other than those we were able to adjust for) that make them less susceptible to succumbing from GvHD (e.g., there might be a higher proportion of type 1-diabetics in the group with diabetes without obesity).

Our study was limited by the dichotomous definition of pre-transplant diabetes (having diabetes requiring treatment with insulin or oral hypoglycemics, but not diet alone). This hindered an analysis of potential differences in transplant outcomes within the patient group with diabetes (as well as a comparison between type 1- and type 2-diabetes, which we did not have data on), which is likely to be heterogenous with regards to the degree of diabetes-induced organ damage and adherence and use of anti-diabetic medications. Similarly, we were limited by our dichotomous definition of pre-transplant obesity (BMI ≥ 30 kg/m^2^), in addition to the fact that the BMI per se is an imprecise measure of fat content, nor does it account for sex and racial differences in fat content and distribution [35]. Moreover, while we included several prognostic covariates in our multivariable analysis, we cannot exclude the possibility of residual confounding in the associations found between obesity and diabetes and increased NRM and between diabetes and lower OS after grade II–IV acute GvHD.

In conclusion, our findings suggest that having obesity or diabetes prior to allogeneic HCT does not influence the risk of developing grade II–IV acute GvHD. However, pre-transplantation obesity and/or diabetes increased the risk of NRM and having diabetes prior to transplant was associated with an increased risk of dying after being diagnosed with grade II–IV acute GvHD. Randomized clinical trials are needed to establish whether an improved glycemic control in patients with diabetes who develop acute GvHD after allogeneic HCT can improve their survival.

## Data Availability

Data and code are available upon request from the corresponding author.
